# Intelligent Posture Training: Machine-Learning-Powered Human Sitting Posture Recognition Based on a Pressure-Sensing IoT Cushion

**DOI:** 10.3390/s22145337

**Published:** 2022-07-17

**Authors:** Katia Bourahmoune, Karlos Ishac, Toshiyuki Amagasa

**Affiliations:** 1 Graduate School of Systems and Information Engineering, University of Tsukuba, Tsukuba 305-8577, Japan; 2 Faculty of Engineering and Information Technology, University of Technology Sydney, Sydney, NSW 2007, Australia; karlos.ishac@uts.edu.au; 3 Center for Computational Sciences, University of Tsukuba, Tsukuba 305-8577, Japan; amagasa@cs.tsukuba.ac.jp

**Keywords:** posture recognition, applied machine learning, IoT, pressure sensing, human well-being

## Abstract

We present a solution for intelligent posture training based on accurate, real-time sitting posture monitoring using the LifeChair IoT cushion and supervised machine learning from pressure sensing and user body data. We demonstrate our system’s performance in sitting posture and seated stretch recognition tasks with over 98.82% accuracy in recognizing 15 different sitting postures and 97.94% in recognizing six seated stretches. We also show that user BMI divergence significantly affects posture recognition accuracy using machine learning. We validate our method’s performance in five different real-world workplace environments and discuss training strategies for the machine learning models. Finally, we propose the first smart posture data-driven stretch recommendation system in alignment with physiotherapy standards.

## 1. Introduction

With recent advances in medical and health sciences and the accessibility of information about healthy living, the awareness of the importance of well-being at work is rising. However, the stresses and commitments associated with modern living can hinder efforts towards achieving better health and well-being on a daily basis. In particular, the increase in desk-bound work and the use of computers and hand-held devices such as smartphones and tablets has exacerbated the problems of sedentary lifestyles and poor sitting posture [1]. A systematic review based on accelerometry measurement of 11 large-scale population studies found that adults spend approximately 10 h a day or approximately 65–80% of the day performing sedentary behaviors [2]. Common sedentary behaviors that most people engage in daily occur while working, commuting, and many leisure activities that require prolonged sitting. Slouching in particular has been termed “the new smoking” [3]. Slouching while sitting is a state where the person’s posture is imbalanced forward or to the sides in addition to any combination of rounded shoulders, forward head posture, and angled neck or lumbar. A vast body of research has shown that poor sitting posture and prolonged sitting lead to a wide range of physical and mental health issues such as lower back pain, neck pain, headaches, respiratory and cardiovascular issues, digestive issues, and an overall higher risk of disease and death [4,5,6,7]. It also contributes to multiple mental health issues such as poor mood, fatigue, low productivity, and depression [8,9]. Furthermore, the lockdown measures introduced by many governments in response to the COVID-19 pandemic led to a surge in working from home (WFH), which had a serious impact on the sedentary and sitting habits of remote workers. While many workplaces are well-equipped with ergonomic chairs and desks, many home settings are far from ideal for prolonged sitting and working from home. Moretti et al. (2020) reported that this lack of ergonomic office furniture in working from home settings may be linked with poorer posture and the onset of musculoskeletal disorders (MSDs) [10].

Addressing the problems of poor sitting posture and prolonged sitting more rigorously is needed to alleviate the impact of their associated health risks on individuals and their economic footprint from lost productivity and national health spending. In addition, to encourage the adoption of an upright posture, occupational health awareness programs often include incentives for workers to stand up, take small and frequent breaks, and perform regular stretching. Frequent postural transitions and regular stretching are important aspects of good posture awareness. Recent studies have shown that incorporating stretching exercises in the training programs of office workers is effective in preventing MSDs in the long term and reducing pain and discomfort [11,12]. Stretching at the workplace has further been found to increase flexibility, prevent injuries due to muscle strain, and improve personal perception of attractiveness and confidence [13]. Therefore, to ensure the adoption of good sitting posture habits, it is important to actively correct poor sitting posture, reduce the amount of time spent sitting down and the amount of time spent in various types of slouching positions in particular, and integrate frequent breaks of activity and proper stretching. Evidently, there are quite a number of factors to actively keep track of, especially when performing other tasks that require focus, which is why we propose a solution using the LifeChair, an Internet of Things (IoT) cushion for real-time posture and activeness tracking.

Various systems have been previously proposed to monitor sitting posture in order to encourage adopting an upright posture with both passive approaches (ergonomics, materials, and fabrics) and active approaches (IoT and sensors). Passive solutions include ergonomic chairs, cushions, elastic bands, and foot rests. Active solutions track sitting posture and include smart cushions, wearable point trackers, and smartphone applications. Recent advances in Artificial Intelligence (AI) and ubiquitous sensing have highlighted the practicality and effectiveness of collecting and mining human-health-related data in real-time for the assessment and improvement of human health and well-being [14]. Better sensing technologies and the large quantities of data they generate have facilitated the application of machine learning to detect and monitor various problems related to well-being such as poor sitting posture. Real-time sitting posture recognition and prolonged sitting monitoring are challenging tasks that require accurate tracking of sitting posture and seated behavior. Sitting is a dynamic task that comes with a wide range of inter-individual variability in body characteristics and differences in working environments, sitting habits, and various other user-specific parameters, which current active posture tracking solutions have yet to address. Moreover, the lack of a standard source of sitting posture and seated behavior data hinders progress in research to achieve active and accurate sitting posture monitoring. Accurate posture tracking leads to effective feedback for active posture correction and good sedentary habits’ promotion. The empowerment of human well-being through posture tracking and correction has important benefits in many domains including the workplace, personal fitness, driver assistance, and entertainment.

In this work, we propose an active posture training solution based on a combination of machine learning and full-back pressure sensing using an IoT cushion called LifeChair for both sitting posture and seated stretch recognition. Our main contributions are as follows:

- We designed an experimental setup for collecting real-world sitting posture and seated stretch pose data from a diverse participant group using a novel pressure sensing IoT cushion.
- We built sitting posture and seated stretch databases that comprise real-time user back pressure sensor data using an active posture labeling method based on a biomechanics posture model and on user body characteristics’ data (BMI).
- We applied and compared the performance of several machine learning classifiers in a sitting posture recognition task and achieved an accuracy of 98.82% in detecting 15 different sitting postures, using an easily deployable machine learning algorithm, outperforming previous efforts in human sitting posture recognition. We were able to correctly classify many more postures than in previous works that targeted on average between five and seven sitting postures.
- We applied and compared the performance of several machine learning classifiers in the seated stretching recognition tasks and achieved an accuracy of 97.94% in detecting six common chair-bound stretches, which are physiotherapist recommended and have not been investigated in related works. While previous works focused on sitting posture recognition alone, we extend our method to include specific chair-bound stretches.
- In the context of AI-powered device personalization, we show that user body mass index (BMI) is an important parameter to consider in sitting posture recognition and propose a novel strategy for a user-based optimization of the LifeChair system.
- We also demonstrate the portability and adaptability of our machine-learning-based posture classification in five different environments and discuss deployment strategies for handling new environments. This has not been investigated by previous works that focus on a single use case of their proposed systems. We demonstrate the impact of local sensor ablations on the performance of the machine learning models in sitting posture recognition.
- We propose, to the best of our knowledge, the first posture data-driven stretch pose recommendation system for personalized well-being guidance.

The rest of this paper is organized as follows: Section 2 reviews the related works in sitting posture monitoring and machine-learning-based sitting posture recognition; Section 3 details our proposed framework for intelligent posture training using machine learning for sitting posture and stretch pose recognition based on a pressure sensing IoT cushion; Section 4 presents the results and discussion of our machine-learning-based sitting posture and stretch pose recognition in the context of the aforementioned contributions; Section 5 concludes this paper with a summary and our future work.

## 2. Related Works

Posture monitoring and correction have received special attention in the last few years, and many different types of posture monitoring devices have been proposed. Broadly, two main types of posture monitoring devices can be found in the scientific literature and in the industry: passive posture training devices and active posture training devices. Passive posture training devices rely on ergonomics and materials to act as add-ons to chairs that target a specific body part of the user to promote a healthy posture form. These include ergonomic chairs, cushions, elastic bands, and foot rests, for example, MTG’s style, Better Back, and Backpod [15,16,17], the Embody Chair by Herman Miller, and ReGeneration by Knoll [18,19]. However, these types of passive posture training solutions have significant shortcomings due to their lack of sensing capabilities and rigidity. They also do not guarantee that users adopt a good posture, as they may still slouch while using them or sit for too long, unaware of their poor posture.

Active solutions aim to address these issues by tracking sitting posture with active components such as sensors and software. They include smart cushions, wearables, point trackers, cameras, robots, and smartphone applications. However, the active solutions available today share many shortcomings with the passive solutions and have limited sensing capabilities, inaccurate posture detection mechanisms, and ineffective feedback schemes. Other cushion-type solutions such as Darma, Cushionware, or e-Cushion have been proposed to track sitting posture using a bottom-rest pressure sensing interface [20,21,22]. These solutions disregard many important aspects of posture training and focus on a basic indication of the balance of pressure as the users sit on them. They can also be uncomfortable and unreliable as they dislocate easily from their optimal position as the user sits on them throughout the day. Wearable posture solutions target body tilt and orientation using accelerometers or gyroscopes. Examples include Upright Go, Waiston, or Lumo Lift [23,24,25]. However, these solutions do not account for full-posture tracking and neglect critical postures such as the forward head posture and rounded shoulders. They are also invasive and intrusive to the user, often requiring direct skin contact or appearance changes. Moreover, these active systems face challenges related to data quality, which hinders their scalable deployment and integration with modern approaches for posture modeling and detection such as machine learning and edge computing. Previous studies in human activity recognition (HAR) have explored the application of machine learning in human sitting posture recognition. Various types of data have been considered for applying machine learning techniques to detect a user’s sitting posture, including camera recording data, depth sensor data, accelerometer and gyroscope data, strain sensor data, and pressure sensing data. Computer vision for human pose detection is a well-established sub-domain of HAR, and many studies have proposed using depth image processing to capture a visual snapshot of the user’s front and machine learning for classifying poses [26,27,28]. However, vision-based methods for posture recognition are limited by field of view constraints, interference and occlusion, sensitivity to lighting conditions, and motion artifacts, in addition to many issues relating to privacy invasion and user trust, which hinder widespread deployment. Wireless methods such as radio frequency identification (RFID) have also been used to detect passive sitting postures, but remain as proof-of-concept solutions prone to inaccuracies in real-world scenarios, in addition to raising important privacy challenges [29].

In broader HAR studies, Anguita et al. (2012) used support vector machines (SVM) for activity recognition, with accelerometers from a mobile phone, and achieved an accuracy of 89% [30]. Wu et al. (2012) found that k-nearest neighbors (k-NN) achieved the best accuracies in detecting different activities such as sitting, walking and jogging, among others, based on iPod touch sensor data [31]. However, these studies target human poses that are significantly divergent from each other such as standing, sitting, stooping, kneeling, and lying down and do not address finer pose sub-classes within each pose such as different sitting postures. Cerqueira et al. (2020) used inertial data from IMUs mounted on a garment to detect six main static upper-body postures using various machine learning models including quadratic SVM, kNN, and linear discriminant analysis (LDA) [32]. The postures targeted in this work do not include sitting postures and only represent broad tilts in the upper body or arm position. Earlier studies achieved fair accuracies in detecting sitting postures ranging from 78% to 88% using principle component analysis (PCA), hidden Markov models, and naive Bayes (NB) [33,34,35]. Table 1 summarizes the key recent works in sitting posture recognition using user sensing and machine learning. These works share many limitations such as proof-of-concept solutions not designed for real-world use and sensing interfaces that do not account for full-posture tracking. Furthermore, the machine learning models they apply for posture recognition are not suitable for real-time deployment due to their computational intensiveness. Furthermore, the datasets they use for training the machine learning classifiers are limited in size and user group diversity. They also target a limited range of sitting postures that do not reflect the dynamicity of seated behavior.

Roh et al. (2018) used a low-cost load cell system made of four load cells mounted on the bottom rest of a chair [36]. They explored using SVM, LDA, quadratic discriminant analysis (QDA), NB, and random forest. They achieved an accuracy of 97.20% with SVM with an RBF kernel on a weight sensor dataset obtained from guided experiments.

Zemp et al. (2016) trained several machine learning classifiers on data obtained from 20 pressure sensors mounted on a chair and on the arm rests in addition to accelerometers, gyroscopes, and magnetometers attached to the rear of the backrest [37]. They trained several machine learning classifiers including SVMs, multinomial regression (MNR), NN, and random forest on manually labeled sensor data obtained from guided experiments to detect seven sitting postures. Their best-performing algorithm was random forest boosted by bagging and ensemble techniques, achieving an accuracy of 90.9%.

A study by Ma et al. (2017) used 12 textile pressure sensors mounted on the bottom rest and backrest of a wheelchair and implemented J48 trees, SVM, multilayer perceptron (MLP), NB, and k-NN to classify five wheelchair-specific sitting postures [38]. They achieved an accuracy of 99.51% using J48 decision trees. However, they used more sensors than in our study and detected only five wheelchair-specific postures.

Hu et al. (2020) used six flex sensors mounted on a regular chair and artificial neural networks (ANNs) implemented on a field programmable gate array (FPGA) to detect seven basic sitting postures with an accuracy of 97.78% with a floating-point evaluation and 97.43% accuracy with the 9 bit fixed-point implementation [39].

Luna-Perejón et al. (2021) used force-sensitive resistors (FSRs) mounted on the bottom rest of a chair and ANN to classify seven sitting postures with an accuracy of 81% [40].

Jeong et al. (2021) combined FSRs and distance sensors embedded in an office chair to detect 11 sitting postures using k-NN and achieved an accuracy of 59%, 82%, and 92% using the pressure sensors only, distance sensors only, and mixed sensor systems, respectively [41].

Farhani et al. (2022) used FSRs attached to the seat pan of a Formid dynamic chair and RF, SVM, and GDTs to classify seven basic sitting postures with an accuracy of around 90% [42].

Stretch pose detection has received little attention in the literature especially using methods such as machine learning. Li et al. (2021) applied a badge-reel stretch sensor to detect spinal bending or stretching. However, this sensing interface is invasive and uses a rigid and simple displacement change computation for spine stretching detection [43].

Previous studies have pointed to a potential effect of user BMI on recognition performance when using pressure sensors to detect sitting posture [38,44]. However, none of these studies fully investigated the importance of BMI in posture recognition, and they showed conflicting results regarding its impact, as detailed in Section 4.2. We investigated the impact of taking BMI into consideration in posture recognition and discuss its importance in the LifeChair in Section 4.2.

Notably, our proposed system for posture monitoring outperforms the works discussed above and is based on a sensing interface that is not fixed—it is non-invasive, portable, and lightweight and can be fit to various chairs for active posture recognition. Our machine learning models are built around data obtained from a biomechanics-based model that covers all areas of the user’s back, including the shoulders, lumbar regions, center of the back, and bottom of the neck. This accounts for critical points of posture monitoring such as vertical and horizontal pressure symmetry, shoulder contact, lumbar contact, in addition to neck and head position, which have been neglected in related works to varying degrees. Furthermore, the type of data collected by our system is also directly relevant to many aspects of user well-being from posture to behavior and suitable for the design of posture data-driven tools for improving posture habits such as personalized stretch recommendation systems.

## 3. Materials and Methods

**Figure 1:**
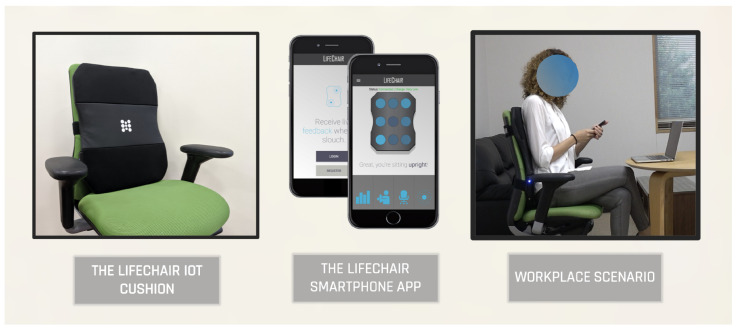
The LifeChair posture training system: The LifeChair IoT cushion (**left**) and the LifeChair smartphone app (**center**) in a workplace scenario (**right**).

In this study, we implemented an IoT cushion called LifeChair to collect sitting posture and seated stretch pose pressure sensing data and applied supervised machine learning techniques to recognize human sitting postures and stretch poses for improving human well-being. The LifeChair is a smart cushion for the backrest of the chair that uses a novel pressure sensing technology specifically developed for human posture detection as detailed in Ishac and Suzuki (2018) and Bourahmoune and Amagasa (2019) (Figure 1) [45,46]. The LifeChair aims to solve the sedentary problem by actively recognizing and correcting the user’s posture to improve his/her health, mood, and productivity. While the design and implementation of the LifeChair device were covered in the works of Ishac and Suzuki (2018) and Bourahmoune and Amagasa (2019), this work expands on key aspects of the posture training system of the LifeChair IoT cushion that relate to the application of machine learning techniques for sitting posture recognition and seated stretch pose recognition in addition to their application for personalized well-being improvement. In this section, we detail the posture monitoring system, data collection protocol, active labeling, and machine-learning-based methods for posture and stretch recognition. We also detail the experimental settings for the portability study and our proposal for the posture–stretch recommendation system.

### 3.1. Sensing Interface

**Figure 2:**
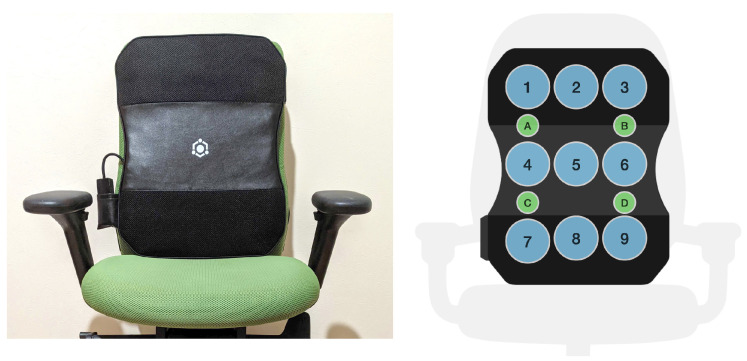
Front view of the LifeChair interface (**left**) and the LifeChair sensor layout (1 to 9) (**right**).

The LifeChair uses specially developed e-textile pressure sensors for tracking the user’s back pressure data [45]. The LifeChair sensors provide force-sensitive output and generate pressure data in the range of 5 g to 10 kg. The 9 sensors are designed to cover the shoulder and lumbar region to account for full-back posture tracking (Figure 2). To capture a wide range of data from users with varying body types and sizes, the size of the sensors is based on the 5th percentile human adult U.S. female (lower limit) and 95th percentile human adult U.S. male (upper limit) seated shoulder width, height, and hip width. The LifeChair cushion model used in this work weighs less than 500 g and measures 52 cm (h) × 40 cm (w) × 2.2 cm (t). The weight and dimensions of the LifeChair cushion make it a portable and flexible solution for posture monitoring in the workplace and at home. The cushion is strapped to a standard office chair through either a single strap or a double strap, depending on the type and shape of the back of the office chair. The LifeChair cushion is also wireless and battery-powered with a 3.7 V lithium-ion battery that provides one to two weeks’ operation time on a single charge. The LifeChair system records in real-time the raw pressure sensor values at a frequency of 5 Hz in addition to the timestamp, posture labels, and user-input data, which include body characteristics (height and weight) and back-pain history data.

**Figure 3:**
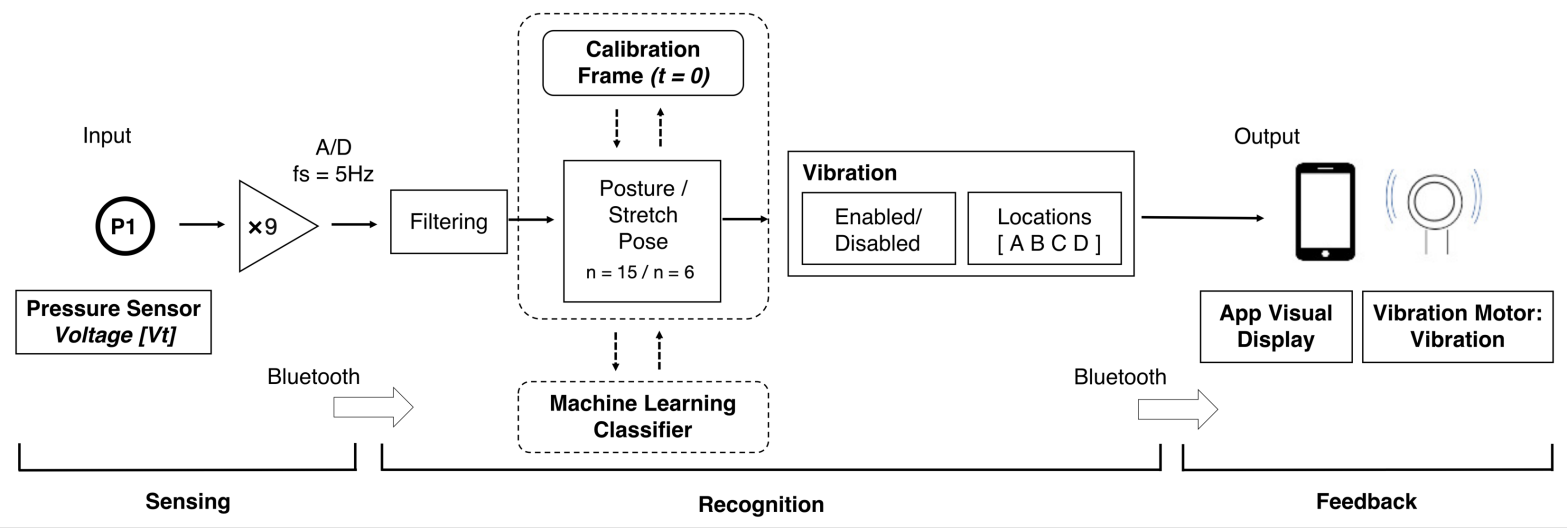
Workflow of our proposed system for machine learning-based sitting posture recognition using the LifeChair system.

The LifeChair system uses bi-directional communication between the IoT cushion and a dedicated smartphone application available for Android and iOS. As described in Ishac and Suzuki (2018), the cushion sends the back pressure data in real-time to the smartphone application via Bluetooth Low Energy (BLE 4.0) [45]. The cushion captures a one-time calibration of the individual upright posture for each user through an application-guided calibration routine and runs a balance check before validating the calibration snapshot. After successful calibration, the device begins to track sitting posture in real-time by matching real-time back pressure sensor readings to sitting postures according to a threshold-based posture template model [45]. This threshold-based method generates posture labels systematically and actively to train the supervised machine learning classifiers for the sitting posture recognition task (Section 3.3). Figure 3 shows the workflow of our proposed method and an overview of the LifeChair system control protocol comprising the sensing phase, the recognition phase, and the feedback phase. The recognition phase combines both the posture template method and machine learning to recognize postures. The LifeChair IoT cushion uses haptic feedback as vibrotactile cues to encourage upright sitting posture, reduce slouching, and alert the users with various reminders. The LifeChair IoT cushion used in this study incorporates four vibration motors and eight different vibration patterns (Figure 2). The haptic feedback patterns are pulsed at a 50% PWM frequency and produce approximately a 3.0 G force of vibration. Each actuator is located towards the corner of each quadrant of the LifeChair to align with the right shoulder, left shoulder, right lumbar, and left lumbar regions of the user. The combination of motors activated notifies the user of how to fix his/her posture. For example, if the upper motors are activated, then the user needs to correct his/her lumbar posture. The combination of vibration patterns informs the user how to fix his/her posture through using haptics in a comprehensible manner without the need for a visual or auditory interface. The quality and effectiveness of the posture correction feedback thus depend on an accurate tracking and recognition of the sitting postures. The LifeChair app provides additional active visual feedback on the main screen, showing a live pressure distribution heat map and the recognized sitting posture.

### 3.2. Sitting Posture and Stretch Pose Data

**Figure 4:**
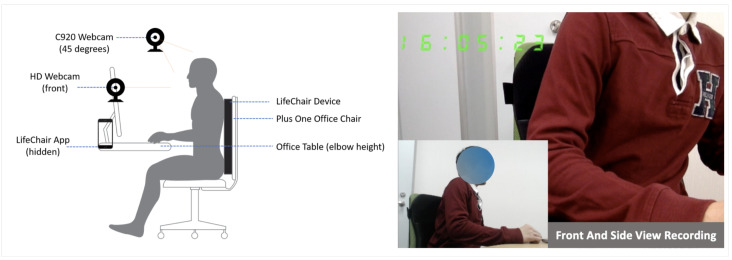
Overview of the experimental setup of sitting posture and stretch pose data collection.

**Posture data**: To build the main sitting posture dataset, 18 healthy subjects (12 male and 6 female) with an average height of 169.07 ± 7.03 (std) and a weight average of 62.50 ± 10.71 (std) belonging to three different groups of BMI (high BMI, normal BMI, low BMI) were instructed to sit in a standard office chair equipped with a LifeChair device and perform a set of common predefined postures. The source of the dataset used in this work is 80% bigger than in our previous work in Bourahmoune and Amagasa (2019) [46]. The chair used for this dataset is the Plus Office Chair Be KD-MA61SL YG, which we denote as the “Standard Back” chair in the portability study. A front-facing camera and a 45-degree front-facing camera were also used to capture video footage of the experiments for visual cross-reference (Figure 4). All experiment subjects were properly coached on how to use the LifeChair, and a one-time calibration was performed for each subject prior to the experiment. All subjects were familiarized with each posture prior to the experiment and were asked to follow an automated slide-show of the postures with no further feedback or instructions in order to capture inter-individual variability in sitting posture. Two rounds were performed where each posture was held for 10 s and repeated three times with an interval of 10 s of upright posture between each posture. The final posture dataset thus consisted of user-input data, timestamps, raw sensor values, and the posture labels (312,587 recordings). The posture labels were one of the following: Upright, Slouching Forward, Extreme Slouching Forward, Leaning Back, Extreme Leaning Back, Left Shoulder Slouch, Right Shoulder Slouch, Left Side Slouch, Right Side Slouch, Left Lumbar Slouch, Right Lumbar Slouch, Rounded Shoulders, Forward Head Posture, Slight Correction Needed, and No User (i.e., no contact with the LifeChair).

**Stretch pose data**: As mentioned in Section 1, integrating frequent stretching while sitting is an important aspect of developing healthy sedentary habits. We propose in this study to incorporate seated stretches into the posture monitoring system by recommending to the users a set of various physio-recommended seated stretches to perform in accordance to their individual sedentary habits and posture trends. We hypothesized that it is possible to capture and detect seated stretch poses in addition to the sitting postures detected by the LifeChair system. We applied machine learning to recognize six different seated stretch poses in order to recommend a stretch guidance routine to the user and check whether the stretches are performed correctly using pressure mapping over time. Within the same experimental setup of the sitting posture data collection, we collected the stretch pose data of six common chair-bound stretches from 13 healthy subjects (8 male and 5 female) with an average height of 169.87 ± 7.37 (std) and an average weight of 62.70 ± 9.50 (std). We specifically targeted stretches that are mostly associated with desk-bound work and easy to perform while sitting down to limit the barriers to performing stretching and introducing activeness to the user’s daily routine. Similar to sitting posture data collection, the users were instructed to perform six common stretches while sitting down on the chair, and each stretch was held for 10 s and repeated three times with intervals of upright posture between each stretch.

### 3.3. Machine-Learning-Based Posture Recognition

In order to build an AI-based agent for sitting posture and stretch pose recognition, we trained multiple supervised machine learning classifiers using the sitting posture dataset and the stretch pose dataset. A core challenge in human activity recognition in general and in sitting posture recognition in particular is the lack of relevant and sufficient annotated training data [47]. In the case of sitting posture recognition, previous works employed a manual labeling method of the sitting position as interpreted by the experimenters. In the research of Roh et al. (2018), a systematic posture data annotation based on the left–right balance and front–back balance of the user weight was implemented [36]. Although we believe this effort is in the right direction, it is incomplete and falls short in accounting for the critical descriptors of sitting posture such as shoulder positions, vertical symmetry, and horizontal symmetry. In this study, we implemented the LifeChair’s innate posture model as an active posture labeling method for training the machine learning classifiers in the posture recognition task. The LifeChair posture model comprises threshold-based posture templates that represent 15 different sitting postures that take into consideration important posture aspects that were not addressed in previous studies (e.g., rounded shoulders, forward head posture). Overall, using the threshold-based posture template method, real-time pressure data values at each individual sensor were compared to the calibration snapshot of each of these individual sensors, and the instantaneous squared difference between these values was assessed against a pre-defined threshold value to determine the current posture according to a state lookup table. This method is described in detail in Ishac and Suzuki (2018) and Bourahmoune and Amagasa (2019), and we expand on it below with how it is used for actively training the machine learning classifiers in the posture recognition task [45,46].

Before tracking sitting posture for a given user, the system first calibrates the subject’s upright posture through an app-guided calibration routine. The LifeChair app then scans the calibration snapshot for approximate balance in pressure distribution from the center-line and alerts the user if an imbalanced calibration is detected. The calibration sensor data pressure readings are then stored as a reference frame to which real-time deviations in pressure are compared. To teach the machine learning models the different sitting postures, the posture labels are generated through the threshold-based posture model, which matches real-time pressure sensor data to posture templates based on the deviation of the errors from the calibration reference array. This posture model takes into account $V_{i}\left(t=0\right)$, which denotes the sensor pressure values at the time of calibration for a given user at each pressure sensor $f_{i}$, where *i* is in reference to the sensor position in the LifeChair IoT cushion shown in Figure 2, $V_{i}\left(t\right)$, which denotes the instantaneous sensor pressure values at time *t*, and *E*, which denotes the deviation errors as defined in Equation (3).

(1) $$V_{i}\left(t=0\right)=\left[f_{1}\left(0\right),\ldots ,f_{9}\left(0\right)\right]$$

(2) $$V_{i}\left(t\right)=\left[f_{1}\left(t\right),\ldots ,f_{9}\left(t\right)\right]$$

(3) $$E{\left(t\right)}_{i}={\left(V_{i}\left(t\right)-V_{i}\left(0\right)\right)}^{2}>\alpha$$

where α in Equation (3) is a constant strictness threshold defined in previous work, which controls the strictness of the error deviation from the calibration reference [45]. The participant is assumed to be upright if the error deviation is smaller than α at each location. If the error exceeds α, a posture is matched based on a state look-up table, as detailed by [45]. This method can detect up to 15 different sitting postures or states, which are then used to train the machine learning classifiers: Upright, Slouching Forward, Extreme Slouching Forward, Leaning Back, Extreme Leaning Back, Left Shoulder Slouch, Right Shoulder Slouch, Left Side Slouch, Right Side Slouch, Left Lumbar Slouch, Right Lumbar Slouch, Rounded Shoulders, Forward Head Posture, Slight Correction Needed, and No User (i.e., no contact with the LifeChair). Over time, the machine learning models trained on this labeled dataset have the ability to capture finer trends in sensor data divergence for posture and recognition than the threshold-based method alone.

As mentioned in Section 2, user body characteristics’ information such as weight and height data is of particular importance in sitting posture tracking and recognition. The LifeChair system also records user body mass index (BMI), which is used as an additional feature in training the machine learning models. The BMI combines information on the user’s weight and height and is defined as the ratio of the weight (kg) to the height squared (m^2^), as shown in Equation (4). We specifically used the BMI as an index of user body characteristics’ data because (1) it captures enough indication about the user’s overall weight, height, and shape and (2) it is a widely used measure of body shape and health that users are familiar with. We thus investigated the impact of including the BMI as a feature for training on the performance of the machine learning classifiers in the posture recognition task.

(4) $$\mathrm{BMI}=\mathrm{weight}/{\mathrm{height}}^{2}$$

**Figure 5:**
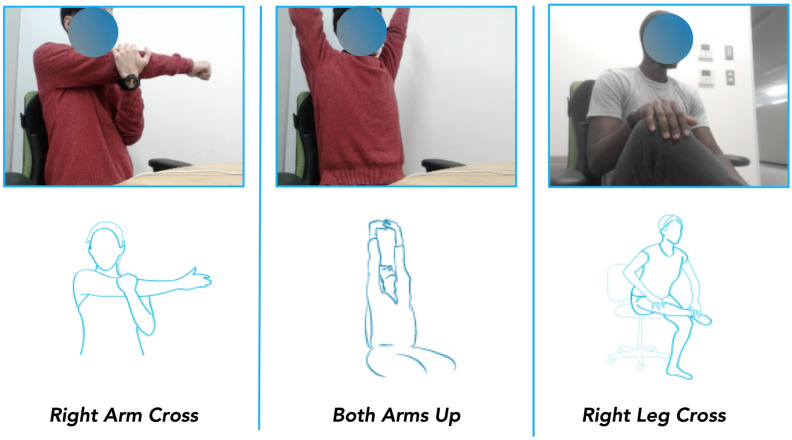
Examples of stretches preformed by experiment participants. Right Arm Cross, Both Arms Up, and Right Leg Cross.

For labeling the stretch pose data, the nature of the stretch pose must be taken into account. Unlike sitting posture, which is a dynamic event, a stretch pose is a static event that requires a conscious and dedicated effort from the user. Subjectivity in stretch interpretation and variable physical predisposition to perform the stretches result in greater inter-individual variability compared to the sitting posture recognition task. A stretch template approach using a similar threshold-based method to the posture recognition task in this case is not desirable. The stretch pose labels were thus obtained manually with video cross-validation and were: both arms up (BAU), hanging arms down (HAD), left arm cross (LAC), right arm cross (RAC), left leg up and across (LLU), and right leg up and across (RLU). This lends further insight into the performance of the machine learning classifiers when trained on data labeled using the posture model described above or with manually labeled data. Figure 5 shows three examples of the stretches performed by the participants, the right arm cross stretch, the both arms up stretch, and the right leg cross stretch.

Ideally, a good machine learning model for this problem should be: (1) highly accurate and (2) computationally cheap and (3) efficiently deployable on smartphones/tablets in real-time. First, achieving a high accuracy is especially important for the LifeChair system because the device provides haptic feedback to the user based on the user’s detected posture. Accurate recognition is key to establishing an effective posture correction scheme and smooth user–device interaction. Second, the posture recognition task should be achieved with a computationally cheap algorithm because the LifeChair aims to track and recognize sitting posture in real-time. The LifeChair app displays in real-time the user’s dynamic pressure distribution on the main app screen alongside the current recognized posture. Finally, it is critical to implement algorithms that guarantee efficient deployment to and integration with various software destined for smartphones and tablets. As noted in Tomasev et al. (2020) [14], despite the appeal of complex machine learning architectures, models with minimum overall complexity are preferred in applications such as the one presented in this work because of their accessibility and suitability for fast deployment. We investigated in our work a variety of linear and non-linear supervised machine learning models that fit these criteria and include: decision trees-classification and regression trees (DT-CART), Random Forest (RF), k-nearest neighbors (k-NN), linear regression (LR), linear discriminant analysis (LDA), naive Bayes (NB), and neural network-multilayer perceptron (MLP). The models were implemented in Scikit-learn using the default hyperparameter settings, unless otherwise specified [48]. For both the posture and the stretch recognition tasks, these models were trained on the raw pressure sensor data, posture label, or stretch label, in addition to the BMI where relevant. K-cross-validation was used for the statistical estimation and comparison of the performance of the models. To investigate the importance of the BMI for posture recognition, group-specific training on each user BMI group was performed [49].

### 3.4. Portability Study

**Figure 6:**
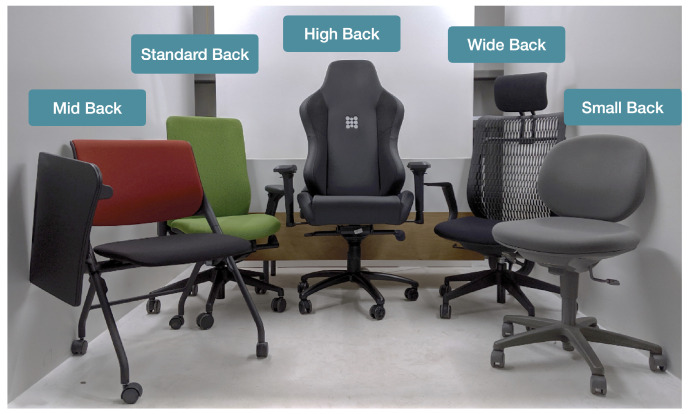
The five different shair types used in the portability study.

To investigate the portability of our machine-learning-based posture recognition system and its adaptability to different environments, we conducted a portability study where we tested the various machine learning models on five datasets obtained from five different chair types. The chair types investigated cover the most common variations in backed chairs and were: the *Small Back* chair type, where the back area covers a smaller area of the user’s back, namely the lumbar and center region with no coverage of the shoulder region; the *Standard Back* chair type, which is the average ergonomic chair with coverage along most of the shoulder region, central region, and lumbar region of the back; the *Mid Back* chair type, which covers the central region only with no coverage of the shoulder region and lumbar region; the *High Back* chair, which has additional coverage vertically in both the shoulder region and the lumbar region, but is curved inwards at the edges; the *Wide Back* chair, which has additional coverage horizontally in the shoulder region, the central region, and the lumbar region, but is slightly curved at the edges. The different chairs used are shown in Figure 6.

As the environment dictates the types of postures that can be adopted, we selected to investigate the recognition of nine common postures using our method for each chair type, and the postures selected were: Forward Head Posture, Leaning Back, No Left Side, No Left Shoulder, No Right Side, No Right Shoulder, Rounded Shoulders, Slouching Forward, Upright. The Forward Head Posture and No Shoulders Posture in particular received little investigation overall in previous works and in particular in settings involving variable environments. In this experiment, ten participants were requested to perform the nine different sitting postures on each chair type. A final portability dataset for every type was compiled for training the different machine learning models to recognize the sitting posture and comparing the performance of the machine learning models when group-trained on every chair type dataset individually or when trained on a global dataset combining the data from all chair types.

### 3.5. Posture–Pose Similarity Assessment

Frequent stretching is an important aspect of good posture habits. We propose to build a stretch pose recommendation system that suggests stretch poses to be performed based on each individual user’s sitting posture trends. In the LifeChair system, the raw sensor data captured for stretch pose and sitting posture is of the same nature: a nine-dimensional vector of raw pressure sensor values corresponding to the back pressure distribution of the user. We propose to use the degree of *dissimilarity* between a stretch pose and a posture as a basis for designing a stretch recommendation system optimized for each user. Since the purpose of performing a given stretch pose is to offset the effect of the prolonged adoption of a given notably different posture, a key idea proposed is to recommend performing a stretch pose that is the least similar to the dominant posture of the user for a target time period. We used the cosine similarity measure to assess the degree of similarity between postures and stretch poses [50]. Specifically, the cosine similarity measures the similarity between two non-zero vectors by calculating the cosine of the angle between them, as shown in Equation (5), where p and s are the sitting posture vector and the stretch pose vector, respectively. An example of its use in applied machine learning is the assessment of the degree of similarity between text documents. This metric is a measurement of orientation and not magnitude, and it can be viewed as a comparison between posture and pose pressure distributions in a normalized space. A high cosine value implies that the stretch pose’s pressure distribution is closely related to that of the sitting posture it is compared to. A low cosine value implies that the stretch pose’s pressure distribution is divergent from that of the sitting posture it is compared to. Based on the calculation of the cosine similarity between every stretch with every posture, it is possible to recommend the stretch pose that has the lowest cosine similarity with the most common posture recorded for a relevant interval of time.

(5) $$\cos\left(p,s\right)=\frac{ps}{∥p∥∥s∥}=\frac{\sum_{i=1}^{n}p_{i}s_{i}}{\sqrt{\sum_{i=1}^{n}{\left(p_{i}\right)}^{2}}\sqrt{\sum_{i=1}^{n}{\left(s_{i}\right)}^{2}}}$$

## 4. Results and Discussion

### 4.1. Sitting Posture Recognition

We present the results of the sitting posture recognition task using multiple machine learning classifiers. In Table 2, we show the performance of: k-nearest-neighbors (k-NN), random forest (RF), naive Bayes (NB), decision trees (DT-CART), linear regression (LR), Linear discriminant analysis (LDA), and neural network-multilayer perceptron (NN MLP) on the augmented posture dataset described in Section 3.2. For each classifier, we report two different results according to the input data used; “Sensor Only” indicates that the input features consisted of the raw sensor values only, and “Sensor + BMI” indicates that the input features consisted of the raw sensor values and user BMI. The scoring metric used for comparison is the overall accuracy as defined in Equation (6). This metric takes into account both the model’s precision and recall where $T_{p}$ is the true positives, $T_{n}$ is the true negatives, $F_{p}$ is the false positives, and $F_{n}$ is the false negatives.

(6) $$\mathrm{Accuracy}=\frac{\left(T_{p}+T_{n}\right)}{\left(T_{p}+T_{n}+F_{p}+F_{n}\right)}$$

As in Bourhamoune and Amagasa (2019) [46], the Random forest classifier and the decision tree classifier achieved the best accuracies in both cases when using sensor data only and sensor data with the participant’s BMI. Random forest achieved the highest accuracy of 98.82% with the combination of sensor data and the BMI as the input and 97.09% with the sensor data only (Table 2). This difference in classification accuracy when using the BMI as an additional feature is significant (Wilcoxon signed rank test, p<0.01) and shows that the BMI can indeed be useful in capturing individual variability in posture adoption.

**Figure 7:**
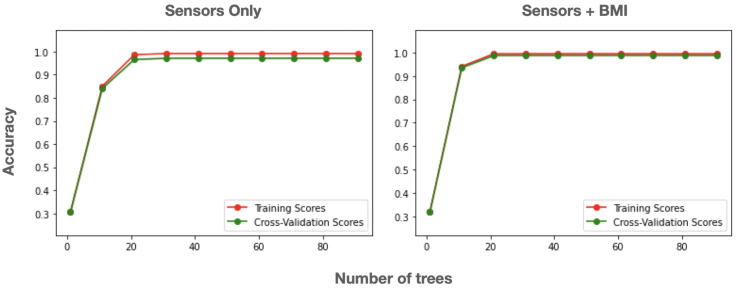
Validation curve (accuracy) for random forest in the sitting posture recognition task when using sensor data only (**left**) and when using sensor data and BMI (**right**).

Random forest is a widely used and popular machine learning algorithm with extensive applications in both the scientific literature and in industry projects [51,52]. Figure 7 shows the validation curves for the random forest classifier when using sensor data only and when using sensor data with the BMI. The maximum accuracy was reached early at a depth of 30 trees, which is a reasonably low threshold for successful real-time use. This result is important because of its suitability for implementation and deployment in mobile and IoT environments.

Zemp et al. (2016) achieved an accuracy of 90.9% using random forest in classifying seven postures [37]. Roh et al. (2018) achieved an accuracy of 97.20% in classifying six postures using RBF-kernel SVM, which is notably too computationally intensive for real-time deployment in IoT edge devices [36]. Zhu et al. (2003) achieved an accuracy of 86% using slide inverse regression in classifying ten postures [53]. Previous studies have achieved fair accuracies ranging from 78% to 88% using PCA, hidden Markov models, and naive Bayes [33,34,35]. Hu et al. (2020) used ANN and achieved an accuracy of 97.78% on a significantly smaller dataset [39]. Luna-Perejón et al. (2021) used FSRs and ANN to achieve an accuracy of 81% [40]. Jeong et al. (2021) used FSRs and distance sensors and k-NN and achieved an accuracy of 59%, 82%, and 92% using the pressure sensors only, distance sensors only, and mixed sensor systems, respectively [41]. Finally, Farhani et al. (2022) used force-sensitive resistors (FSRs) and random forest and achieved an accuracy of 94% [42].

Our results outperformed these works and the previous studies that attempted to combine pressure sensing and machine learning for posture recognition. It is important to point out that these studies are optimized for the datasets obtained in their respective experiments, and a direct comparison of the algorithms’ performance in classifying sitting posture is not the goal of this work. In our proposed system as well, the results were optimized for the postures generated by the LifeChair’s active labeling method, and one of our goals was to find the best classification models for the sitting posture recognition task with the LifeChair IoT cushion. Ma et al. (2017) achieved an accuracy of 99.51% using J48 decision trees [38]; however, they used more sensors than in our study and detected only five wheelchair-specific postures. The machine-learning-based LifeChair system detected 15 different different postures with a high accuracy using a common, fast, and robust machine learning classifier. The improvement in classification accuracy achieved in this study is most likely due to the combination of the spatial deployment of the sensors in the LifeChair interface and the biomechanics-based data labeling and training of the machine learning models.

This is important because poor interface design and poorly modeled human activity data are significant challenges in HAR. In this study, these two challenges were effectively addressed by implementing a sensing interface specifically designed to capture a wide range of posture-related parameters such as full-back balance tracking and shoulder and lumbar region contact monitoring with a validated biomechanics-based human posture model. Furthermore, we were able to detect two key postures related to the head and neck position that previous studies did not directly address. Users can maintain full-back balance and full contact with the LifeChair device, but exhibit neck and shoulder poses that are detrimental to their health in the long run. These postures are the forward head posture (Slight) and rounded shoulders (No Shoulders), which are widely common today due to the rise in extended interaction with hand-held devices.

To further investigate this impact of the spacial distribution of pressure sensing data on the performance of the best-performing classifier for posture recognition, we report the results of the sensor ablation study (Table 3). We conducted three types of sensor feature ablations: nine individual ablations for each sensor, three horizontal ablations for sensor groups distributed horizontally, and three vertical ablations for sensor groups distributed vertically. For the individual ablations, the lowest accuracy was reported for sensor 7, sensor 3, sensor 6, sensor 5, and sensor 1, in that order, with 95.40% for sensor 7. This suggests that the areas covered by these sensor affect the accurate recognition of the postures investigated in this study, which is consistent with the intended spatial design to capture the extreme sways in positions for each posture. Interestingly, the horizontal ablations had more impact on the classification accuracy than the vertical ablations. The lowest accuracy reported across all of the ablations was for the group of sensor 4, sensor 5, and sensor 6, with 88.05%, which lies in the center of the sensing interface and captures data on the left and right sways in position, in addition to the maintenance of contact in the center. In contrast, the vertical ablations had less impact on the classification accuracy with accuracies ranging between 92.42% and 92.88%. These results point to the importance of the spatial distribution of the pressure sensors for capturing salient information on the back pressure distribution when used as an input for machine learning classifiers for posture recognition. Notably, across all the different types of sensor ablations, despite the missing features, either individually, horizontally, or vertically, the range of accuracies for posture recognition using the LifeChair remained comparable to or higher than the similar works discussed in Section 2.

### 4.2. BMI Divergence

Table 4 shows the classification accuracy of the best-performing machine learning classifier for three different participant groups based on their BMI. The posture recognition accuracy using random forest when performed separately for each group showed that the accuracy for low-BMI users was significantly lower than that of normal-BMI users and that of high-BMI users (Wilcoxon signed rank test, p<0.01). In the study of Ma et al. (2017), the BMI had no effect on the accuracy of the machine learning models in posture detection, while Kim et al. (2018) noted a lower accuracy for smaller children in their child posture study [38,44]. In the case of Ma et al. (2017), the absence of a BMI effect might be due to the position of the sensors (bottom rest of a wheelchair) and the limited range of motion associated with wheelchair-specific postures. Height is an important parameter to consider in sitting posture. The majority of office chairs have height adjustment features to control the elevation of the bottom rest from the ground; this is because user height has a direct effect on the user’s weight distribution on the chair and, consequently, on the user’s posture and level of comfort. Weight is an important parameter in our system because our pressure sensors are force-based. Thus, one of the ways to improve performance in sitting posture recognition tasks is to include the BMI as a feature in addition to the sensor data. Another way to address this divergence in our system lies in the initial posture model itself. In the system presented in this study, a potential source of this difference is the threshold α in Equation (3), which took a uniform value for all three groups during the calibration step for our data collection experiments. As α represents the strictness of the sensor error deviation from the calibration reference, the low-BMI group might require a less strict value than the normal-BMI group and the high-BMI group. Therefore, to improve the accuracy for the low-BMI group and, thus, the overall accuracy of the system, we combined the inclusion of the BMI as an input feature for training the machine learning classifiers and the implementation of a lower α upon calibration for users with a BMI lower than 18.5 kg/m${}^{2}$. This effectively allows for a user-based personalization and improvement of the system centered around their body characteristics’ data.

### 4.3. *Portability and Adaptability*

Table 5 shows a summary of the model accuracies for posture recognition in the portability study. For each chair type, two results are shown: the accuracy of the model when group-trained on its respective dataset and the accuracy of the model when trained on the global dataset that includes all chair types. One aim of this portability study is to explore the adaptability of the machine-learning-based posture recognition in variable environments. This is important because the portability and adaptability of machine-learning-based sitting posture sensing systems are some of the main limitations of these solutions.

Across all chair types, the accuracy is higher when training the model on the individual chair type’s group dataset as compared with training the model on the dataset of all chair types combined. For the group training mode, the accuracies ranged between 97.19% and 98.29%, with the highest accuracy recorded for the Wide Back chair type. This is likely due to the fact that the Wide Back chair type provides a full surface area for the LifeChair IoT cushion with all sensor groups covered both horizontally and vertically. Due to the spatial distribution of the pressure sensors embedded in the LifeChair cushion, the shape and position of the backrest of the chair may have an impact on the performance of the posture recognition models. However, this impact can be alleviated through multiple strategies at the device settings’ level and on the model training level. On the device level, environment-based calibration solves this problem by calibrating the reference pressure distribution on a given chair for computing the threshold-based method used to train the machine learning classifiers. On the model training level, a group training or global training strategy of the machine learning models can be considered to improve the robustness of the machine learning models when deployed.

For the global training mode, the accuracies ranged between 56% and 92% with the Mid Back chair type recording the lowest recognition accuracy. This drop in accuracy for the Mid Back chair type in the global training mode is consistent with our observations in the horizontal sensor group ablations in Section 4.1, whereby the accuracy was shown to be most sensitive to ablating the horizontal sensor groups. The group training for the Mid Back chair type recorded a higher accuracy of 97.19%, suggesting that in cases where the environment is unique (Mid Back chair type, i.e., double horizontal sensor group ablation), training the model on a the specific environment dataset is more robust than using a globally trained dataset.

As the global training recorded high accuracies for all chair types except the Mid Back chair type for the reasons explained previously, we can adopt a deployment strategy for new environments that starts with a globally trained model, then transitions into a specific group-trained model for the new environment. This is useful for cases when new environment data are scarce where a globally trained model can be used to produce fair to high posture recognition accuracies until a sufficient amount of data is obtained for this new environment. The impact of this is two-fold: first, this allows for building an individualized posture recognition model with better recognition accuracy for each environment; second, this contributes to the robustness and generalizability of the global model.

### 4.4. Seated Stretch Recognition

**Figure 8:**
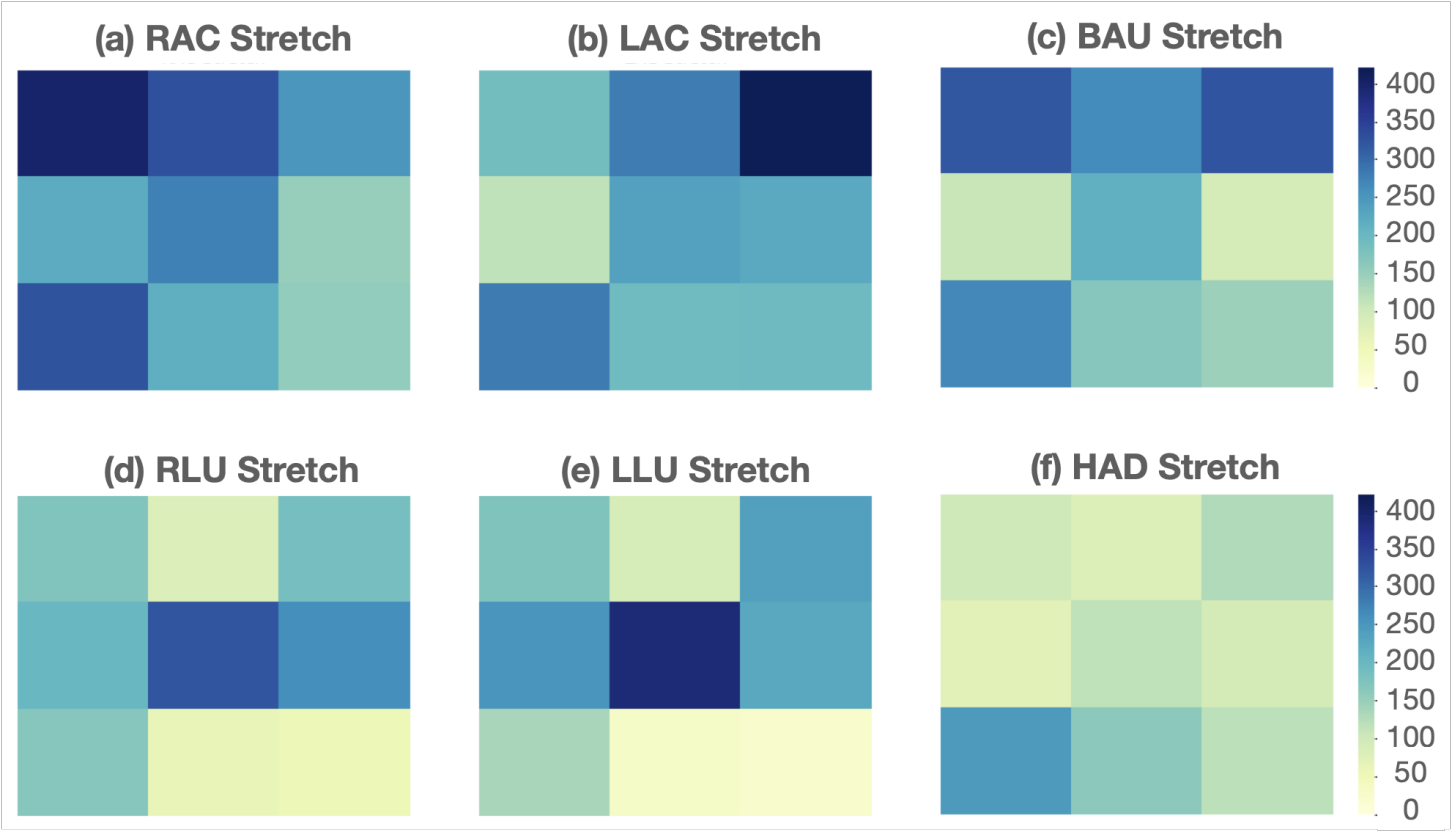
Average pressure distribution heat maps of the six common chair-bound stretches: (**a**) Right Arm Cross (RAC); (**b**) Left Arm Cross (LAC); (**c**) Both Arms Up (BAU); (**d**); Right Leg Up (RLU); (**e**) Left Leg Up (LLU); (**f**) Hanging Arms Down (HAD).

Figure 8 shows the heat maps of the average pressure distribution for each of the six stretches across all subjects. A low pressure reading is represented in the lighter greens, and a high pressure reading is represented in the darker blues. For example, Figure 8a, which represents the heat map for the stretch pose “right arm cross” (RAC), shows a high pressure reading on the upper left sensor. This captures accurately the pose performed by the participants where they extended the right arm across the chest and pressed the left arm on the right elbow. The pressure distribution heat maps show that the six stretches are visually distinguishable from each other. On this basis, we trained the same machine learning classifiers used in the posture recognition task to recognize these six stretches and report their performance results in Table 6. Based on the results from the posture recognition and BMI divergence experiments, the input used for the stretch recognition task consisted of the sensor data and user BMI. Consistent with our results from the posture recognition task, random forest performed the best in recognizing the six stretch poses with an accuracy of 97.94%.

**Figure 9:**
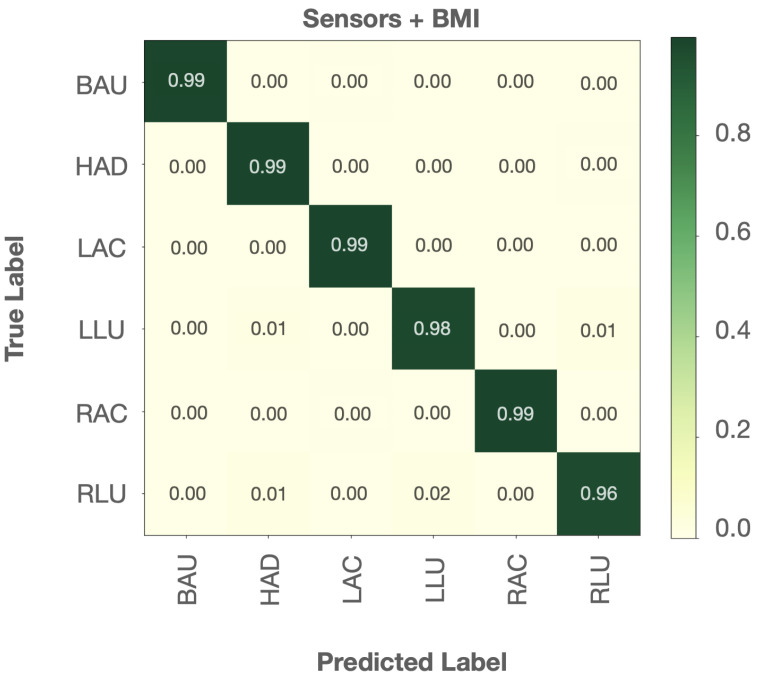
Normalized confusion matrix for the Random Forest classifier in the stretch pose recognition task.

Figure 9 shows the confusion matrix for the stretch pose recognition task. Each stretch pose investigated was correctly classified with an accuracy between 96% and 99%. It can be observed that the stretch pose where the participants had their right leg up and across (RLU) was the most prone to misclassification. RLU was mostly misclassified as the stretch pose where subjects were leaning forward with their arms hanging down (HAD) or as the stretch pose where subjects had their left leg up and across (LLU). A possible explanation for this is related to the subjects’ sitting behavior around the lumbar region. As can be seen on the heat maps in Figure 8, all subjects on average had a consistent reading on the ninth sensor positioned behind their left lumbar region. A similar pattern was noticed in a previous study that used the LifeChair system for the validation of its haptic feedback correction [45]. In that study, the pressure distribution of the participants in a LifeChair feasibility experiment with and without the vibration feedback showed that when using the LifeChair without correction or vibration feedback, the innate posture distribution was on average imbalanced with a high reading on the left lumbar region and a low reading on the right shoulder region. This might be due to a higher representation of right-hand-dominant participants who display a compensation for a lower pressure on the right shoulder with a higher pressure on the left side of the back in general and the left lumbar region in particular [54]. This discrepancy could be potentially illustrating a trend captured by the machine learning recognition of the stretch poses that could not be picked up on by the threshold-based method alone.

### 4.5. Posture–Stretch Recommendation System

**Figure 10:**
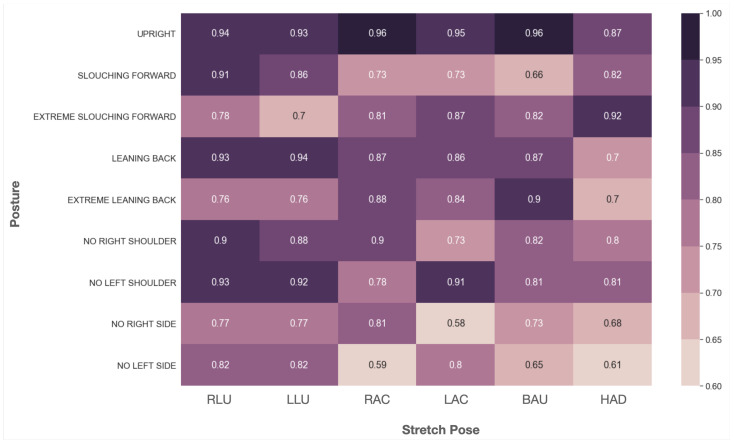
Cosine similarity matrix between the pressure distribution of the sitting postures and the stretch poses.

Figure 10 shows the cosine similarity matrix between the six seated stretches and nine common sitting postures that are representative of the aforementioned 15 posture labels with a focus on imbalances along the left and right axes and the forward and backward axes. A high cosine value indicates that a stretch pose’s pressure distribution is closely related to that of a sitting posture. By comparing the cosine similarity between each stretch and each posture for all possible pairs, it is possible to establish a ranking system that matches each sitting posture with its *least-similar* stretch pose. These results show that, indeed, our posture–stretch recommendation method was successful in capturing the correct stretch pose for each posture in alignment with physiotherapy standards. For instance, for the “Upright” posture, the least similar stretch pose is “Hanging Arms Down” (HAD); for the Slouching Forward posture, the least similar stretch pose is “Both Arms Up”, followed equally by both “Right Arm Cross” (RAC) and “Left Arm Cross” (LAC); for the posture “Extreme Slouching Forward”, which notably engages the lumbar region, the least similar stretch is “Left Leg Up” (LLU) and “Right Leg Up” (RLU); for the postures “Leaning Back” and “Extreme Leaning Back”, the least similar stretch pose is “Hanging Arms Down” (HAD); for the postures “No Right”, “No Left”, “No Right Shoulder”, and “No Left Shoulder”, their least similar stretches are those with their opposite position to the posture, respectively, i.e., for “No Right” and “No Right shoulder”, it is “Left Arm Cross” (LAC), and for “No Left” and “No Left Shoulder”, it is “Right Arm Cross”.

Thus, the proposed posture–stretch matching approach effectively captured an intuitive relationship between the six stretch poses and their matched postures. The cosine similarity was able to acquire salient information about the divergence between the sitting posture vector and the stretch pose vector for successful matching. This allows for the introduction of a smart individualized stretch pose recommendation system based on personal posture data that can be tuned to each individual user’s posture performance and sitting habits.

## 5. Conclusions

We proposed a machine-learning-based sitting posture and stretch recognition method using a pressure sensing IoT cushion. We developed an extensive experimental protocol for sitting posture and seated stretch pressure data collection in divergent working environments. We applied multiple machine learning models in a sitting posture recognition task and in a seated stretch recognition task. We achieved 98.82% in recognizing 15 different sitting postures using the random forest classifier, which is a common and accessible machine learning model suitable for deployment in mobile and IoT environments. We also achieved 97.94% in recognizing six popular seated stretches. We showed that user body characteristics’ data (BMI) has a significant impact on the performance of the machine learning model in recognizing sitting posture. These results allowed us to further explore the importance of various local sensor groups for the performance of the machine learning models, and we demonstrated that the horizontal spatial distribution is important for accurate posture recognition using machine learning in our proposed framework. Furthermore, we validated the portability and adaptability of our method in five different chair environments and proposed training strategies for the machine learning models depending on the deployment environment. Finally, we proposed the first posture–stretch recommendation system and showed that our method was successful in capturing salient links between each posture and stretch pose studied.

In future work, we aim to implement our system in various real-world scenarios such as driving, healthcare, and entertainment for improving human well-being. We aim to expand on the portability study, particularly in cases where the contact area in the back of the chair makes data collection more challenging. We also plan to explore the performance of the machine-learning-based posture and stretch recognition using an IoT cushion that covers a different sensing area, such as the bottom rest of the chair or a localized back region.

## Figures and Tables

**Table 1:** Summary of the key related works in sitting posture recognition from sensing data using machine learning.

Sensing Data	Algorithm	Sitting Postures	Reference
Load Cells	SVM, k-NN, LDA, QDA, NB, RF	6	[36]
Pressure Sensors	SVM, NN, RF, MNR	7	[37]
Pressure Sensors	J48 trees, SVM, MLP, NB, k-NN	5	[38]
FSRs	ANN	7	[40]
Flex Sensors	ANN	7	[39]
FSRs and Distance Sensors	k-NN	11	[41]
FSRs	RF, SVM, GDT	7	[42]

**Table 2:** Classification performance of the tested algorithms in the sitting posture recognition task. * p<0.01 Wilcoxon signed rank test.

Algorithm	Sensors Only	Sensors + BMI
RF	0.9709	0.9882 *
DT-CART	0.9619	0.9843
k-NN	0.9213	0.9229
NN (MLP)	0.8009	0.8838
LR	0.5367	0.5520
LDA	0.5316	0.5529
NB	0.4171	0.4830

**Table 3:** Classification performance of random forest classifier in the sitting posture recognition task for different ablation settings.

Ablation Type	Ablated Sensor/s	Accuracy
Individual	1	0.9589
	2	0.9659
	3	0.9549
	4	0.9622
	5	0.9581
	6	0.9575
	7	0.9540
	8	0.9600
	9	0.9600
Horizontal	1, 2, 3	0.8983
	4, 5, 6	0.8805
	7, 8, 9	0.8982
Vertical	1, 4, 7	0.9042
	2, 5, 8	0.9288
	3, 6, 9	0.9042

**Table 4:** Accuracy divergence based on user BMI. * p<0.01 Wilcoxon signed rank test.

User Group	$\mathrm{BMI}(\mathrm{kg}/m^{2})$	Accuracy
Low BMI	BMI < 18.5	0.97001 *
Normal BMI	18.5≤ BMI ≤25.0	0.9898
High BMI	BMI > 25.0	0.9846

**Table 5:** Accuracy of the best-performing machine learning classifier (Random Forest) in the posture recognition task in five different environments using two modes of training.

Environment	Training Mode	Accuracy
Small Back	Global Training	0.7600
	Group Training	0.9801
Standard Back	Global Training	0.9200
	Group Training	0.9772
Mid Back	Global Training	0.5600
	Group Training	0.9719
High Back	Global Training	0.8600
	Group Training	0.9782
Wide Back	Global Training	0.7600
	Group Training	0.9829

**Table 6:** Classification performance of the tested algorithms in the stretch pose recognition task.

Algorithm	Sensors + BMI
RF	0.9794
DT-CART	0.9658
k-NN	0.9143
NN (MLP)	0.8121
LR	0.5780
LDA	0.5526
NB	0.4936
